# Transitioning from child to adult-oriented HIV clinical care for adolescents living with HIV in Ethiopia: results from a retrospective cohort study

**DOI:** 10.11604/pamj.2020.37.13.21407

**Published:** 2020-09-03

**Authors:** Sander Broström, Axel Andersson, Inger Kristensson Hallström, Degu Jerene

**Affiliations:** 1 Department of Health Sciences, Faculty of Medicine, Lund University, Lund, Sweden,; 2 KNCV Tuberculosis Foundation, The Hague, Netherlands

**Keywords:** Ethiopia, adolescents living with HIV, transition

## Abstract

**Introduction:**

Ethiopia has one of the largest number of adolescents living with HIV (ALHIV). As these adolescents reach adulthood they need to transfer from pediatric to adult-oriented clinics. Clear implementation guidelines for transition are lacking and factors associated with successful transition are inadequately investigated. Our objective was to describe the rate and age of transition from child- to adult-oriented care and the factors associated with transition success among ALHIV in selected health facilities in Ethiopia.

**Methods:**

a retrospective cohort study of adolescents was conducted in eight health facilities in two regions of Ethiopia: Addis Ababa and the Southern Nations, Nationalities and Peoples´ Region (SNNPR). The study was embedded within a broader study originally aimed at studying clinical outcomes of adolescents. The proportion of adolescents who transitioned was calculated and the association between baseline characteristics and transition was assessed by bivariate and multivariate analysis.

**Results:**

of 1072 adolescents, 8.7% transitioned to adult care. The most frequent age of transition was 15 (range: 10-22). Multivariate analysis generated two significant findings: adolescents from Addis Ababa were more to likely transitioned than adolescents from SNNPR (aOR: 2.18; 95% CI=1.17-4.06; p<0.01), as well as disclosed adolescents compared to those not disclosed of their HIV-status (aOR: 4.19; 95% CI=1.57-11.98; p<0.01).

**Conclusion:**

transition occurred in less than 10% of participants, in a wide range of age, indicating a lack of implementation policies regarding the transition process. Thereto, we found that adolescents from Addis Ababa and those disclosed of their disease, were more likely to transition. Further studies are needed to better understand factors associated with transition success.

## Introduction

As of end of 2018, a total of 37.9 million people were living with HIV, a majority of which reside in eastern and southern Africa [1]. AIDS-related deaths have dropped by 56% between 2004 and 2018 due to improved access to antiretroviral therapy (ART). Similarly, overall new HIV infections have declined by an estimated 16% between 2010 and 2018. This decline was more pronounced, 41% in children, making adolescents living with HIV (ALHIV), most of which are perinatally infected [2,3], constitute a dramatically larger group than before (currently 1.8 million worldwide) [2]. Ethiopia is one of the countries with the largest ALHIV population [3]. ALHIV have unlike all other age groups not experienced reductions in AIDS-related mortality; to the contrary, the mortality rate in this group has more than doubled between 2000 and 2015 [2]. AIDS-related illness is the predominant cause of mortality among African adolescents [3] and sub-Saharan Africa held 87% of adolescent AIDS-related deaths in 2015 [2]. Furthermore, for independent demographic reasons, a dramatic increase in the number of African youth is estimated and is expected to accompany a challenging increase of new infections among adolescents [2]. Currently, an estimated 25 million adolescents are living in Ethiopia [4]. ART decreases HIV-related morbidity and mortality and prevents transmission, if it is adhered to [5]. Adherence to ART is commonly defined as taking ≥95% of prescribed doses [6,7], as this level of adherence has been demonstrated necessary for viral suppression and positive health outcomes [8].

Adherence levels in Ethiopia are generally high [9], but since only 67% of people living with HIV (PLHIV) are aware of their HIV-status, a mere 51% PLHIV in Ethiopia are virally suppressed [1], predisposing for spreading of the virus. ART has been suggested to be cost effective for use in Ethiopian district hospitals [10]. Adolescence, defined as the age of 10-19 [2], involves the rapid developmental transformation from childhood to adulthood [11]. These biological and psychosocial changes involve risk-taking and experimental behavior, as well as new ways of relating to one´s own future and health [11]. Regarding HIV related knowledge, among Ethiopian 15-19 year olds, 24% of women have comprehensive HIV knowledge (compared to 38% of men) [12]. Higher prevalence of sexual risk behaviors such as low condom use has been observed in ALHIVs, even compared to perinatally HIV-exposed but uninfected adolescents [13]. Further demonstrating the vulnerability of this group, ALHIV experience a high prevalence of internalized stigma of the disease [14]. Initiation of ART of adolescents in Ethiopian decentralized care has (despite poor viral suppression) yielded positive outcomes clinically [15], whereas outcomes for untreated patients in Ethiopia have been detrimental [16]. Achieving high rates of adherence in ALHIV is therefore a key concern. A prerequisite for adherence to medication is successful transition from child to adult-oriented care [17]. However, many adolescents stop participating in health care services during this period [18]. Ergo, the transition process poses a unique challenge for retaining ALHIV in the HIV care cascade.

Highly functional transitional care is therefore of vital importance, reflected in many facilities developing transitioning plans [19]. Nevertheless, these have been insufficiently implemented, mainly due to lack of staff training [19]. Indeed, patients in paediatric care are often poorly prepared for the transition to adult care, forcing caregivers to make special efforts to facilitate the process [19]. Strict age criteria are certainly not always applied by the clinics and the requirements for qualifying for transition vary, resulting in a wide spectrum of transitional age [19]. As of 2014, Ethiopia had not implemented a nation-wide system for transition from paediatric to adult-oriented care, though this was suggested as a possible intervention in the national guidelines for comprehensive HIV prevention, care and treatment [20]. Although guidelines on transitional age seem to be absent, transition at the age of 15 is supposedly the accepted practice in the Ethiopian health care system. Factors associated with successful transition are inadequately investigated and experience with managing the transition process is limited, especially in low-resource settings. The objective of the study was to describe the rate and patterns of transition, as well as the factors associated with transition success, among adolescents living with HIV in selected health facilities in Ethiopia.

## Methods

**Study design and setting:** a retrospective cohort study was conducted in eight health facilities in two regions of Ethiopia: Addis Ababa and the Southern Nations, Nationalities, and Peoples´ Region (SNNPR). Addis Ababa, the capital of the country, has the lowest prevalence of poverty and lowest fertility rate in the country and the highest rate of literacy and media exposure [21]. The median duration of education is more than twice as long in Addis Ababa compared to SNNPR among men and more than six times as high among women [12]. HIV prevalence is more than five times higher in Addis Ababa compared to SNNPR (5.2% versus 0.9%) [22] and high risk heterosexual sex among men is the highest in the country [21]. SNNPR, whose capital is located 250 kilometers to the south of Addis Ababa, is home to around 20% of the Ethiopian population and holds great ethnic and lingual diversity [23]. Cash crops and livestock are important sources of income in the region, which does not suffer the same food and income insecurity as neighboring regions, although many people are poor [23]. SNNPR has the highest prevalence of HIV-stigma in Ethiopia, as compared to Addis Ababa, where acceptance is more widespread [22]; two thirds of 15-49 year olds in SNNPR hold intolerant attitudes towards PLHIV compared to one sixth in the capital [12]. Child mortality rate in SNNPR is more than twice as high as in Addis Ababa, which has the lowest rate in Ethiopia [12]. Seven hospitals and a health center were chosen on the grounds of being high patient load facilities with already established research collaboration with the investigators. All the health facilities render comprehensive HIV care and follow the WHO ART treatment guidelines and the national guidelines for comprehensive HIV prevention, care and treatment.

**Sample size and sampling procedure:** one thousand and seventy two adolescents (aged 10-19 years) living with HIV constituted the study population; 582 from Addis Ababa and 490 from SNNPR health facilities. The sample size was originally determined with the assumption to compare treatment outcomes between adolescents and children. Sample size assumptions are described in a previous study [24]. The adolescent transition study was embedded within a broader study plan.

**Data collection and management:** the data was collected between March and June 2014 for patients enrolled during the period January 1^st^ 2005 through December 31^st^ 2013. Trained nurses assisted by site data clerks collected the data from the patients´ pre-ART and ART registers. Senior pediatricians supervised the data collection. The cohort was originally designed to enable the study of clinical outcomes among adolescent living with HIV. However, two additional variables were included in the analyses in an effort to study adolescent transition patterns: any history of transition and age of transition.

**Statistical analysis:** descriptive statistics were used for the cohort profile, participants´ baseline characteristics and characteristics of transition. Associations between variables and history of transition were assessed by bivariate analysis, done by cross tabulation and logistic regression. Associations with significance <0.25 in bivariate logistic regression qualified for screening in multivariate analysis. In the multivariate logistic regression analysis adjusted odds ratio (aOR), p-value and 95% confidence interval (CI) were used to assess statistical strength of the associations. History of transition from child to adult-oriented clinic was treated as a dichotomous variable. CD4-count at ART initiation and CD4-count pre-ART were also dichotomized, for both of which cut-off was defined as ≥350cells/mm^3^. WHO-stage pre-ART and WHO-stage at ART initiation were dichotomized into less advanced and advanced stages (I-II and III-IV, respectively). In bivariate logistic regression, the dichotomized age of diagnosis variable was also used and age was treated as a continuous variable. We used SPSS for Windows version 24.0 for data analysis. Statistical significance was defined as p<0.05. Ethical considerations: collecting and handling the data was approved in 2014 by the Institutional Review Board of Addis Ababa University, College of Health Sciences; The National Research Ethics Review Committee; The Ethics Review Committees of Addis Ababa and SNNPR Health Bureaus. Before collecting the data from personal medical records, official letters of co-operation were given to the respective hospitals, after which approval of the data collection was granted. Since this was a retrospective cohort study using de-identified data, according to the ethical guidelines in Ethiopia, obtaining patients´ consent was not necessary. Patients´ confidentiality was ensured by using de-identified data. Patients were offered the same care, as prescribed by the national guidelines and the WHO treatment guidelines, irrespective of their participation in the study.

## Results

**Baseline characteristics:** of the 1072 patients analyzed a slight majority were treated in Addis Ababa (54.3%) and most had urban addresses (91.5%) (Table 1). Two participants (1.8%) got their HIV diagnosis before the age of 5, 182(17%) at the age of 6-10, 471(44%) at 11-14 years of age and 417(38.9%) were 15 years or older at diagnosis. Disclosure status was available for 76.8% of participants, 77.9% of which had been disclosed of their HIV status. Age of disclosure was recorded for 502(46.8%) of adolescents. The majority of these were disclosed after the age of 15(286) and the most frequent age of disclosure was 18 years (120). Almost two thirds (66.2%) of the adolescents were in an advanced stage of disease (WHO-stage III or IV) at ART initiation. Hospital youth group participation data were available for 59.3% of participants, of which 29.2% of those applicable participated (Table 1).

**Table 1:** characteristics of adolescents at baseline, adolescent HIV cohort study, Ethiopia

		Noumber (valid%)
**Sex**	Male	427 (39.9)
	Female	644 (60.1)
**Age**	10	189 (17.6)
	11	131 (12.2)
	12	161 (15.0)
	13	97 (9.0)
	14	73 (6.8)
	15	55 (5.1)
	16	40 (3.7)
	17	42 (3.9)
	18	178 (16.6)
	19	106 (9.9)
**Region**	Addis Ababa	582 (54.3)
	SNNPR	490 (45.79
**Residence**	Urban	934 (91.5)
	Rural	86 (8.4)
**Age of HIV diagnosis**	2-5	2 (0.2)
	6-10	182 (17.0)
	11-14	471 (43.9)
	≥15	417 (38.9)
**Disclosure status**	Disclosed	641 (77.9)
	Not disclosed	182 (22.1)
**CD4-count pre-ART**	<350 cells/mm^3^	693 (67.8)
	≥350 cells/mm^3^	329 (32.2)
**CD4-count**	<350 cells/mm^3^	730 (89.8)
	≥350 cells/mm^3^	83 (10.2)
**WHO-stage pre-ART**	I-II	501 (47.9)
	III-IV	544 (52.1)
**WHO-stage at ART initiation**	I-II	275 (33.8)
	III-IV	539 (66.2)
**Hospital youth group participation**	Yes	186 (29.2)
	No	293 (46.1)
	N/A	157 (24.7)

**Figure 1:**
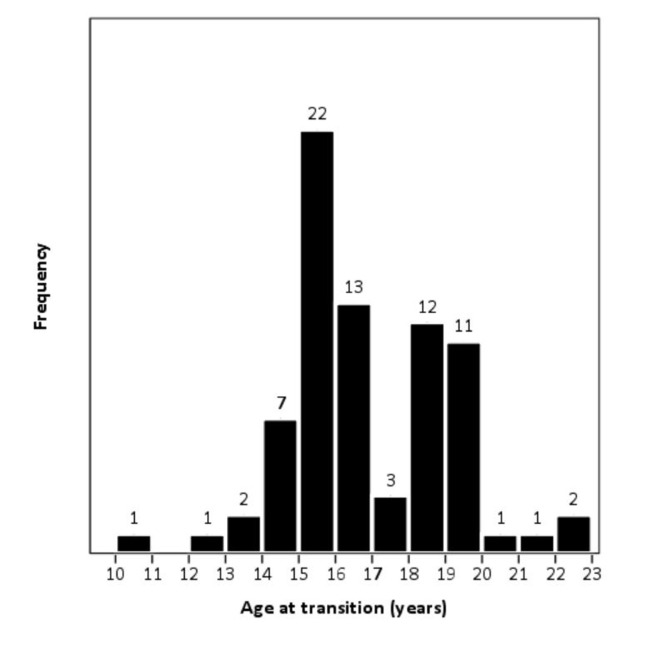
frequency distribution of ages of adolescent transition from child to adult-oriented care

**Characteristics of transition:** transition related information was available for 997(93.0%) study participants, of which 87(8.7%) were reported to have moved to adult clinics. Of these 87 transitioned adolescents, transitional age was available for 76. The most frequent age of transition was 15 years (22 participants), followed by 16, 18 and 19 years (13, 12 and 11 participants respectively) (Table 2). The median age of transition was 16 years (interquartile range, 15-18 years). Ages of transition are illustrated in Figure 1.

**Table 2:** bivariate analyses of factors associated with adolescent transition in HIV care, Ethiopia

Variable		No. transitioned (% within category)	Pearson’s chi2 (p-value)
**Sex**	Male	41 (10.3)	1.982 (0.159)
	Female	46 (7.7)	
**Region**	Addis Ababa	63 (12.4)	17.243 (0.000)
	SNNPR	24 (4.9)	
**Residence**	Urban	83 (9.5)	8.985 (0.01)
	Rural	0 (0)	
**Age of HIV diagnosis**	2-10	9 (5.1)	3.502 (0.061)
	≥11	78 (9.5)	
**Disclosure status**	Disclosed	68 (11.2)	13.064 (0.000)
	Not disclosed	4 (2.3)	
**CD4-count pre-ART**	<350 cells/mm^3^	59 (9.0)	0.003 (0.956)
	≥350 cells/mm^3^	26 (8.8)	
**CD4-count**	<350 cells/mm^3^	74 (10.6)	0.853 (0.356)
	≥350 cells/mm^3^	6 (7.3)	
**WHO-stage pre-ART**	I-II	47 (10.4)	2.863 (0.091)
	III-IV	38 (7.3)	
**WHO-stage at ART initiation**	I-II	35 (13.5)	4.467 (0.035)
	III-IV	45 (8.6)	
**Hospital youth group participation**	Yes	20 (10.9)	0.142 (0.986)
	No	32 (11.2)	

**Factors associated with transition:** the rate of transition was more frequent in boys (10.3%) than girls (7.7%) but the difference was not statistically significant (p>0.1). However, transition was significantly more frequent among adolescents from Addis Ababa compared with adolescents from SNNPR (12.4% versus 4.9%; chi-square=17.24; p<0.001). All of the adolescents who transitioned had urban addresses, significantly associating residence with transition (p=0.01) in this bivariate analysis. Disclosed participants more frequently transitioned to adult care (11.2% versus 2.3%; chi-square=13.06; p<0.001). WHO-stage at ART initiation was significantly more frequent among those initiating treatment in less advanced stages (13.5% versus 8.6%; chi-square=4.47; p<0.05). CD4-count pre-ART, CD4-count at ART initiation, WHO-stage pre-ART and hospital youth group participation were not significantly associated with transition (p>0.05) (Table 2). Bivariate logistic regression identified nine variables qualifying to multivariate analysis (p<0.25): sex, age, region, age of HIV diagnosis, disclosure status, WHO-stage pre-ART and WHO-stage at ART initiation. Residence, CD4-count pre-ART, CD4-count at ART initiation and hospital youth group participation did not qualify to multivariate analysis due to p>0.25. Multivariate logistic regression generated two significant findings: region and disclosure status. Adolescents from Addis Ababa were more likely to transition than adolescents from SNNPR (aOR: 2.18; 95% CI=1.17-4.06; p<0.01) and the same was true for disclosed adolescents compared to those who were not disclosed of their HIV-status (aOR: 4.19; 95% CI=1.57-11.98; p<0.01). Sex, age (treated as a continuous variable), age of HIV diagnosis, WHO-stage pre-ART and WHO-stage at ART initiation were not significantly associated with successful transition (Table 3).

**Table 3:** multivariate logistic regression analysis of factors associated with transition in HIV care, Ethiopia

Variable		aOR (95% CI)	P-value
Sex	Male vs Female	1.023 (0.587-1.781)	0.937
Age	[as a continuous variable]	1.057 (0.945-1.183)	0.330
Region	Addis Ababa vs SNNPR	2.180 (1.169-4.063)	0.014
Age of HIV diagnosis	2-10 vs ≥11	2.308 (0.905-5.891)	0.080
Disclosure status	Disclosed vs Not disclosed	4.193 (1.567-11.984)	0.007
WHO-stage pre-ART	I-II vs III-IV	1.844 (0.752-4.528)	0.181
WHO-stage at ART initiation	I-II vs III-IV	1.132 (0.457-2.806)	0.788

aOR=Adjusted Odds Ratio; CI=Confidence Interval

## Discussion

In the studied cohort 8.7% of the adolescents transitioned from child to adult-oriented care. Age 15 years was the most frequent age of transition. The range of transitional age was 10 to 22 years old. Adolescents disclosed of their HIV-status and adolescents treated in Addis Ababa, were significantly more likely to successfully complete transition to adult-oriented care. The fact that only 8.7% of adolescents transitioned to adult care should spark interest since almost 40% of the participants were 15 years or older at baseline, implicating that many participants transitioned well into adulthood without transitioning to an adult clinic. Plausible explanations for this include: lack of policies on when the transition should occur (or, if any policies exist, lack of health care provider (HCP) compliance), overburdened adult clinics and unwillingness of patients and/or healthcare practitioners (HCPs) to transition patients to adult care. The last-mentioned has previously been described in South Africa, where HCPs at the pediatric clinics reported some reluctance to transition adolescents, as this would end their relationship with the patients [25]. They also felt a need to protect the adolescents from overburdened adult clinics and a setting which they worried would be judgmental and depersonalized [25]. Thereto, it is conceivable that the adolescents are more comfortable with the service providers they have been accustomed to and therefore rather stay at the pediatric clinics.

In addition to the low number of transitioned patients, the wide range of transitional age (10-22) makes the lack of implementation transition plans evident. This is similar to previous findings from sub-Saharan Africa, where the ages of transition have varied between 13 and 22 [19]. Thereto, the transitional ages are not equally distributed in our study, with one peak seen at 15 years and another one at 18 years. Conceivably, this may be attributed to the fact that pediatric care conventionally involves children under the age of 15, as this is often considered the mark of adulthood in a medical sense. The second peak at 18 may be explained by the co-occurrence of legal majority. Being treated in Addis Ababa was significantly associated with higher rates of transition compared to SNNPR. This might relate to the lower level of education in SNNPR and higher levels of poverty, HIV-stigma and discrimination [12], since education [9], poverty [26,27] and stigma [26,28-30] have previously been shown to be barriers of ART adherence in Ethiopia. However, these findings are on adult adherence and the relevance to adolescent transition remains to be settled. Conceivably, too, the higher child mortality rate in SNNPR [12] indicates that the health services are generally less utilized and/or developed, influencing also the rate of transition. Disclosed adolescents were more likely to transition to adult care. Since patients not aware of their HIV-status will naturally not be as prone to take measures to receive HIV-care, this may not be surprising.

For the transition of these patients to occur other persons or entities (such as caregivers, HCPs and the health care system), would need to be proactive in retaining these patients in care. Not disclosing the HIV-status to patients (adolescents in particular) is a well described problem, associated with poor adherence to ART and poor virological outcomes [31]. Barriers to HIV disclosure to adolescents described are: social norms, presumptions of adolescents´ capacity to understand what HIV-positivity implies, hardship in determining the right time for disclosure and importantly stigmatization of the disease [32]. Related, adolescent patients disclosing their HIV to friends and family have been shown to increase social support [26,33], decrease fear of stigma and facilitate adherence to ART [26]. Nonetheless, many adolescents (in the present study over one fifth) remain non-disclosed, perhaps in a misguided effort to protect them. In other words, HIV stigma might indirectly hinder transition, via non-disclosure. Moreover, stigma has been shown to impede transition directly: a previous study in sub-Saharan Africa demonstrated that anticipation of stigma and discrimination at the adult clinics severely hinders the transition process [19]. In Ethiopia these concerns may unfortunately be justified, as inpatients with HIV report HCPs being impolite, fearing to physically contact them, breaching confidential information, delaying services, giving substandard care and even denying them care [30].

Hence, HIV stigma, a well-recognized factor in preventing optimal care in Ethiopia [28-30,34] and worldwide [1,14,19,35], might constitute a key barrier to transition, directly and indirectly. Holistic strategies for adolescent transition to adult care are lacking in sub-Saharan Africa in general [18] and are absent from the Ethiopian national guidelines [20]. An ambitious work by the African Network for Care of Children Affected by HIV and AIDS studied the national policies and guidelines of pediatric and adolescent HIV care in Ethiopia and their implementation. The study found that adolescents were disregarded as a group, as only a pediatric and a general HIV care package existed [36]. Moreover, the guidelines lacked comprehensive schemes to implement adolescent friendly services and to retain adolescents in care [36]. In addition to this, many HCPs did not have comprehensive knowledge of what the guidelines and policies entailed and many felt that policies were inadequate regarding age of disclosure, methods of disclosure and counselling associated with disclosure [36]. An American study stresses the importance of involvement of both adolescent and adult clinics in achieving a continuous HIV-care cascade [37]. In other words, adolescent transition seems to be a neglected area of service in Ethiopia. The strengths of the present study lie in its relatively large sample size and that it uses multivariate analysis to adjust for confounding factors. This is also one of the first reports of adolescent transition in HIV care in an Ethiopian setting and perhaps one of a few globally. The limitations lie in the retrospective nature of the data collection, as this did not allow us to make more accurate estimates for transition rates.

## Conclusion

In this study, we found that transition occurred in less than ten percent of the participants, in a wide range of age, indicating that there is a lack of implementation policies on the transition process. In addition to this, we found that adolescents from Addis Ababa and those disclosed of their HIV-status were more likely to complete the transition process. The need for well-coordinated, functional transitional care is of vital importance, emphasized by the fact that ALHIV is a rapidly growing group. Further studies are needed to better understand the underlying barriers to and facilitators of successful transition to adult care and its impact on treatment outcomes. The beneficial effect of using digital health technologies for improved tracking of transition pathways, for example, is an important area of intervention for further study. We also suggest that adolescents be prioritized as a group in HIV care, on a policy level and that adolescent friendly models be implemented to successfully transition these patients to adult care and equip them with the necessary tools to successfully manage living with this chronic disease.

### What is known about this topic

- High rates of attrition from care is a common challenge among adolescents living with HIV;
- Poorly managed transition from child to adult-oriented care is one of the key contributors to attrition from care.

### What this study adds

- Low rates of transition from child to adult-oriented care among older adolescents suggests lack of clear transition implementation guidelines;
- Adolescents in rural areas are more likely to remain under child-oriented care despite growing to adulthood;
- The study identified areas for further investigation.
